# Tomographic Evaluation of Temporal Bone Pneumatisation with Sinus Variations

**DOI:** 10.3390/diagnostics16213656

**Published:** 2026-11-05

**Authors:** Esra Yeşilova, Esin Kılıç, Muzaffer Bilgin

**Affiliations:** 1 Department of Oral and Maxillofacial Radiology, Faculty of Dentistry, Eskişehir Osmangazi University, Eskişehir 26040, Türkiye; esin.kilic@ogu.edu.tr; 2 Department of Biostatistics, Faculty of Medicine, Eskişehir Osmangazi University, Eskişehir 26040, Türkiye; mbilgin@ogu.edu.tr

**Keywords:** cone-beam computed tomography, temporal bone, paranasal sinuses, pneumatisation, anatomic variability, radiological anatomy

## Abstract

**Background/Objectives:** Temporal bone pneumatisation and paranasal sinus variations have mostly been studied separately. This study evaluated temporal bone pneumatisation with frontal, ethmoid and sphenoid sinus variations using cone-beam computed tomography (CBCT) and investigated relationships among these structures. **Methods:** In this retrospective cross-sectional study (ethics approval no. 2025/58), 88 CBCT scans of patients aged ≥25 years were analysed. Temporal bone pneumatisation, frontal sinus size, International Frontal Sinus Anatomy Classification (IFAC) cells, ethmoid variants and sphenoid sinus pneumatisation were categorised using established classifications. Bilateral concordance and age-, sex- and inter-regional associations were tested with false discovery rate (FDR) correction, and exploratory Firth regression and multiple correspondence analysis were performed. **Results:** The sample comprised 49 females and 39 males (mean age 41.6 ± 13.3 years). Bilateral concordance was good for supra-bulla (κ = 0.76), supraorbital ethmoidal (κ = 0.61) and agger nasi cells (κ = 0.60) and moderate for temporal pneumatisation (κ_w_ = 0.56). The frontal sinus was larger on the left side (FDR-adjusted *p* = 0.002). No age-, sex- or inter-regional association survived FDR correction. In exploratory regression, male sex was associated with frontal septal cells (adjusted odds ratio [OR_adj_] = 3.75, 95% CI 1.36–11.15) and right supraorbital ethmoidal cells (OR_adj_ = 3.48, 95% CI 1.35–9.62). Multiple correspondence analysis revealed no single dominant dimension. **Conclusions:** Temporal bone and paranasal sinus pneumatisation showed marked bilateral concordance, but no inter-regional association survived correction for multiple testing. The sex differences in frontal septal and supraorbital ethmoidal cells are hypothesis-generating and need confirmation in larger samples.

## 1. Introduction

The skull has a well-characterised aeration system that is clearly depicted and readily recognised on radiographic images. The temporal bone, together with the frontal, sphenoid and ethmoidal sinuses, is thought to develop gradually through positive solid tissue pressure transmitted from the nasopharynx via the Eustachian tube opening [1].

The temporal bone is composed of five parts positioned in different planes relative to one another, which makes interpretation challenging for both radiologists and surgeons [2]. Despite the generally well-pneumatised pattern of the mastoid portion, the degree of pneumatisation shows considerable interindividual variability [3].

The frontal sinus is located within the frontal bone and has potential value for personal identification because its shape, size and configuration vary between individuals [4]. The ethmoid bone is centrally located within the sinonasal complex and plays a role in olfaction, humidification, ventilation and resonance. The ethmoidal cells also contribute critically to shaping the drainage pathways of the frontal and maxillary sinuses via the osteomeatal complex [5]. The sphenoid bone is a major component of the skull base; it contains the sphenoid sinus and serves as a conduit for several vital neurovascular structures [6,7].

Accurate assessment of these pneumatised anatomical structures is important for diagnosis, surgical planning, optimisation of patient outcomes and forensic identification [7,8,9,10,11]. Previous studies have examined temporal bone pneumatisation and paranasal sinus variations, reporting sex-related differences, volumetric characteristics and clinically relevant anatomical associations. For example, Mitsea et al. demonstrated larger frontal sinus dimensions in males [12], Sethi et al. highlighted the surgical risks associated with sphenoethmoidal variants such as the Onodi cell [13] and Kim et al. reported a positive correlation between sphenoid sinus pneumatisation and mastoid air cell volume [14]. More recently, Katı et al. reported a significant association between zygomatic process pneumatisation and sphenoid sinus pneumatisation on CBCT [15]. However, temporal bone pneumatisation in conjunction with frontal, ethmoidal and sphenoidal sinus variations has not been evaluated collectively, and studies assessing these structures using three-dimensional imaging techniques remain limited. Assessing these regions together in the same individuals has practical value. Endoscopic sinus and skull base procedures often involve more than one pneumatised compartment, and variants such as the Onodi cell are associated with higher surgical risk [13]. If pneumatisation in one region were related to that in another, a variant seen in one region could prompt a closer look at the others on the same preoperative scan. The present study was designed to test whether such relationships exist and to describe how these variants occur together in a single population.

Cone-beam computed tomography (CBCT) is widely used in oral and maxillofacial radiology for three-dimensional evaluation of maxillofacial structures. CBCT acquires volumetric data using a cone-shaped X-ray beam and a two-dimensional area detector during a single rotational scan, allowing reconstruction of isotropic voxel-based three-dimensional images [16]. By overcoming the limitations of conventional two-dimensional radiography—such as superimposition, distortion and magnification—CBCT provides high-resolution visualisation of osseous craniofacial anatomy. As a result, it has become an essential imaging tool in dental practice, particularly for implant planning, assessment of impacted teeth, temporomandibular joint evaluation, maxillofacial pathology and detailed analysis of paranasal sinus anatomy [17,18].

The aim of this study was to evaluate temporal bone pneumatisation in relation to variations of the frontal, ethmoid and sphenoid sinuses using CBCT scans and to investigate potential relationships among these anatomical features.

## 2. Materials and Methods

### 2.1. Ethical Approval and Study Population

This retrospective study was conducted in accordance with the principles of the Declaration of Helsinki and was approved by the Non-Interventional Clinical Research Ethics Committee of Eskişehir Osmangazi University (approval date: 28 May 2025; approval no. 58). CBCT scans obtained between 2 January 2019 and 15 May 2025 were retrospectively reviewed from the institutional archive. The radiology archive was screened consecutively. Patients were included if they (a) were aged 25 years or older, (b) had images of diagnostically adequate quality and (c) had no pathology in the regions of interest. Patients were excluded if they (a) were younger than 25 years, (b) had artefacts affecting image interpretation or measurement or (c) had pathology in the study area. Images in which any of the evaluated anatomical structures was not fully covered by the field of view were also excluded. The sample size was calculated a priori using G*Power (version 3.1.9.7) for a chi-square test with one degree of freedom, a medium effect size (w = 0.3), a two-sided α of 0.05 and 80% power, which yielded a minimum of 88 patients (actual power = 0.804). Screening stopped once 88 patients meeting the criteria had been identified. The maxillary sinuses were not evaluated because their pneumatisation can change after tooth extraction, in edentulous regions or after loss of an adjacent tooth; the frontal, ethmoid and sphenoid sinuses are not affected by such dental factors.

### 2.2. Imaging Protocol and Evaluation

CBCT scans were acquired using two systems: NewTom VGi (QR s.r.l., Verona, Italy) and Planmeca ProMax 3D Mid (Planmeca Oy, Helsinki, Finland). All scans were obtained for diagnostic purposes using the standard protocols of each device. The same acquisition parameters were used for all patients scanned with each device and were retrieved retrospectively from the archive. The field of view was 24 × 17 cm for the NewTom VGi and 20 × 17 cm for the Planmeca ProMax 3D Mid, and all evaluated anatomical structures had to lie within the scanned volume. Scans were acquired at 110 kVp and 3 mA with a voxel size of 0.300 mm on the NewTom VGi and at 90 kVp and 8 mA with a voxel size of 0.400 mm on the Planmeca ProMax 3D Mid. Image assessments were performed using Planmeca Romexis (version 4.6.R; Planmeca Oy, Helsinki, Finland) and NewTom NNT (version 16.7; QR s.r.l., Verona, Italy) software. All images were evaluated together by two observers with different levels of experience: an oral and maxillofacial radiologist with 27 years of experience and a research assistant and resident in oral and maxillofacial radiology with 2 years of experience. Each classification was assigned by joint decision. Because the subcategories of the classifications used are clearly defined, no disagreement arose that could not be resolved by consensus.

### 2.3. Temporal Bone Pneumatisation

**Figure 1:**
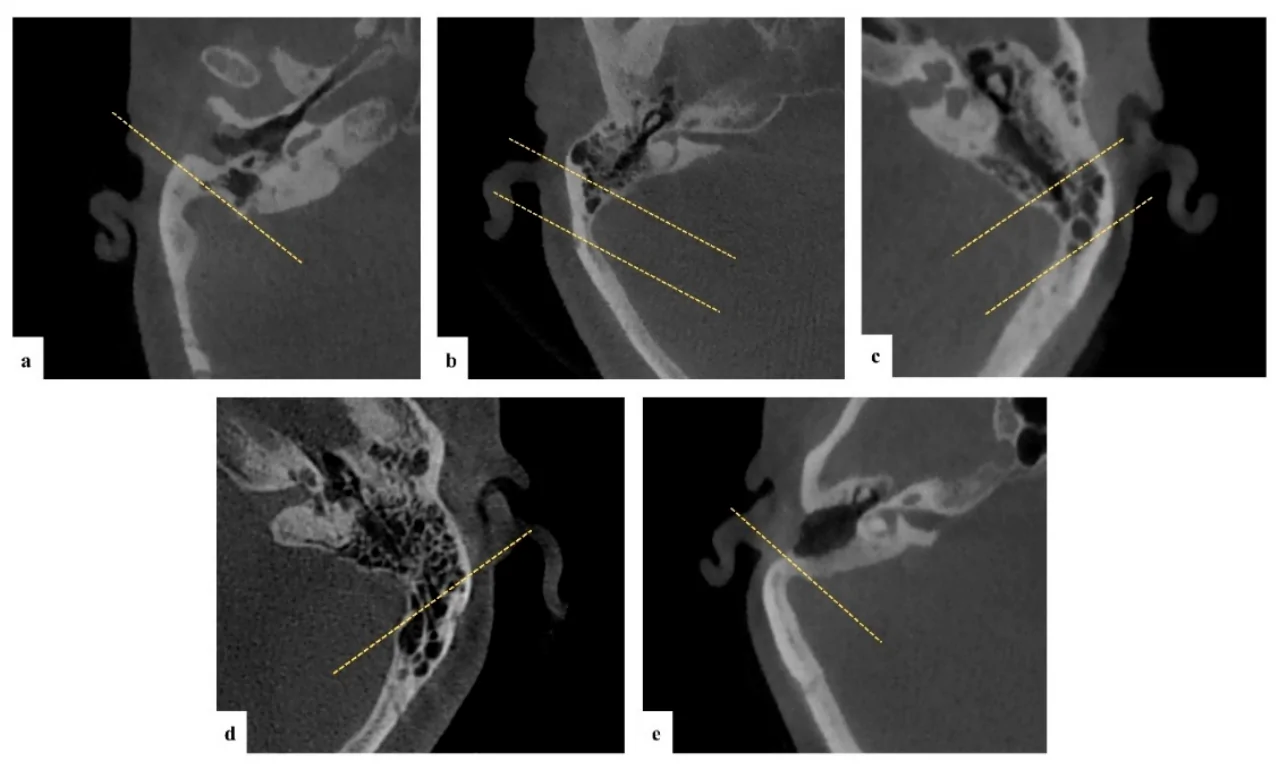
**Temporal bone pneumatisation.** CBCT images showing different degrees of pneumatisation: (**a**) hypopneumatisation; (**b**) moderate pneumatisation; (**c**) good pneumatisation; (**d**) hyperpneumatisation. (**e**) In one of the evaluated patients, no pneumatisation was observed; therefore, an additional category termed the acellular type was added to the classification.

Temporal bone pneumatisation was assessed on axial CBCT images using the classification proposed by Han et al. [19] and subsequently applied by Aladeyelu et al. [20], with the sigmoid sinus (SS) used as the reference landmark. Pneumatisation was graded on a single axial slice at the level defined by Han et al. [19], with the reference lines drawn as described in that study. Pneumatisation was categorised into five groups (Figure 1):

- **Hypopneumatisation:** Pneumatisation limited to the line drawn to the most anterior point of the SS.
- **Moderate pneumatisation:** Extension between the anterior and lateral reference lines of the SS.
- **Good pneumatisation:** Extension between the lateral and posterior margins of the SS.
- **Hyperpneumatisation:** Extension beyond the posterolateral line drawn from the posterior aspect of the SS.
- **Acellular:** Absence of visible air cells (this category was added based on the study findings).

### 2.4. Frontal Sinus Size Evaluation

Frontal sinus size was evaluated on coronal CBCT images using the supraorbital line (SOL) and midorbital line (MOL), as described by Guerram et al. [21].

In the NewTom NNT software, the SOL curve was defined in the multiplanar reconstruction (MPR) 1 view, generating Panorex images with a slice thickness of 0.300 mm, 2 mm spacing and 51 sections. In Planmeca Romexis, the SOL curve was created on the panoramic reformat page using a slice thickness of 0.400 mm.

**Figure 2:**
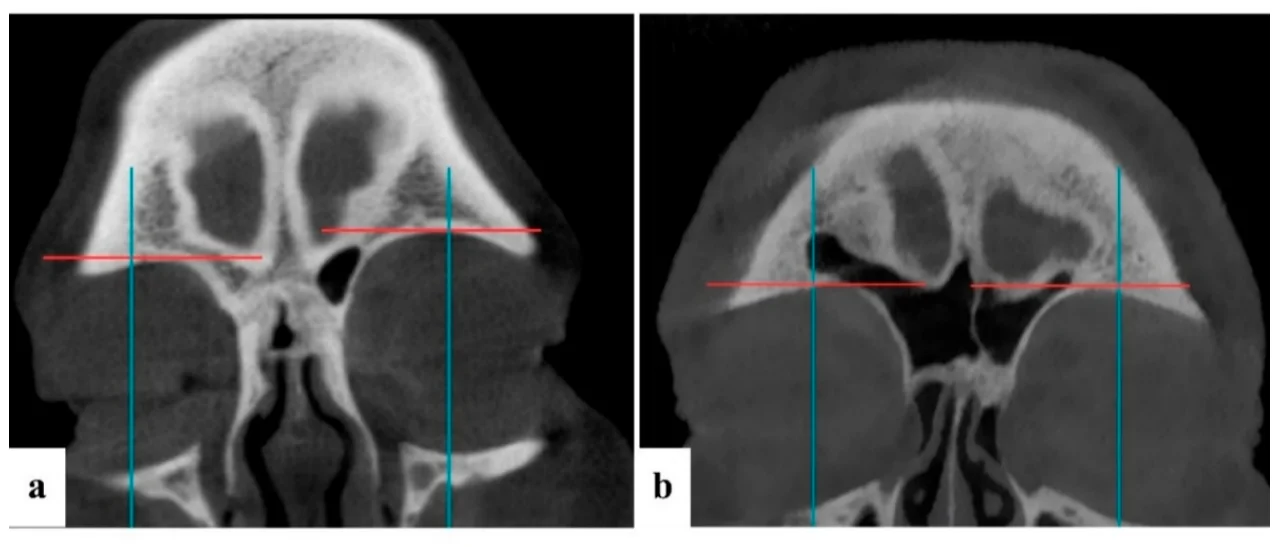
**Frontal sinus size. **(**a**) Coronal CBCT image showing an aplastic frontal sinus on the right side and a hypoplastic frontal sinus on the left side. (**b**) Coronal CBCT image showing a hyperplastic frontal sinus on the right side and a medium-sized frontal sinus on the left side. Red line, supraorbital line (SOL); blue line, midorbital line (MOL).

Frontal sinus size was classified as follows, according to Guerram et al. [21] (Figure 2):

- **Aplasia:** Absence of frontal pneumatisation.
- **Hypoplasia:** Pneumatisation limited to the area below the SOL.
- **Medium size:** Pneumatisation extending laterally but limited by the MOL.
- **Hyperplasia:** Pneumatisation extending laterally beyond the MOL.

### 2.5. Frontal Sinus Variations

**Figure 3:**
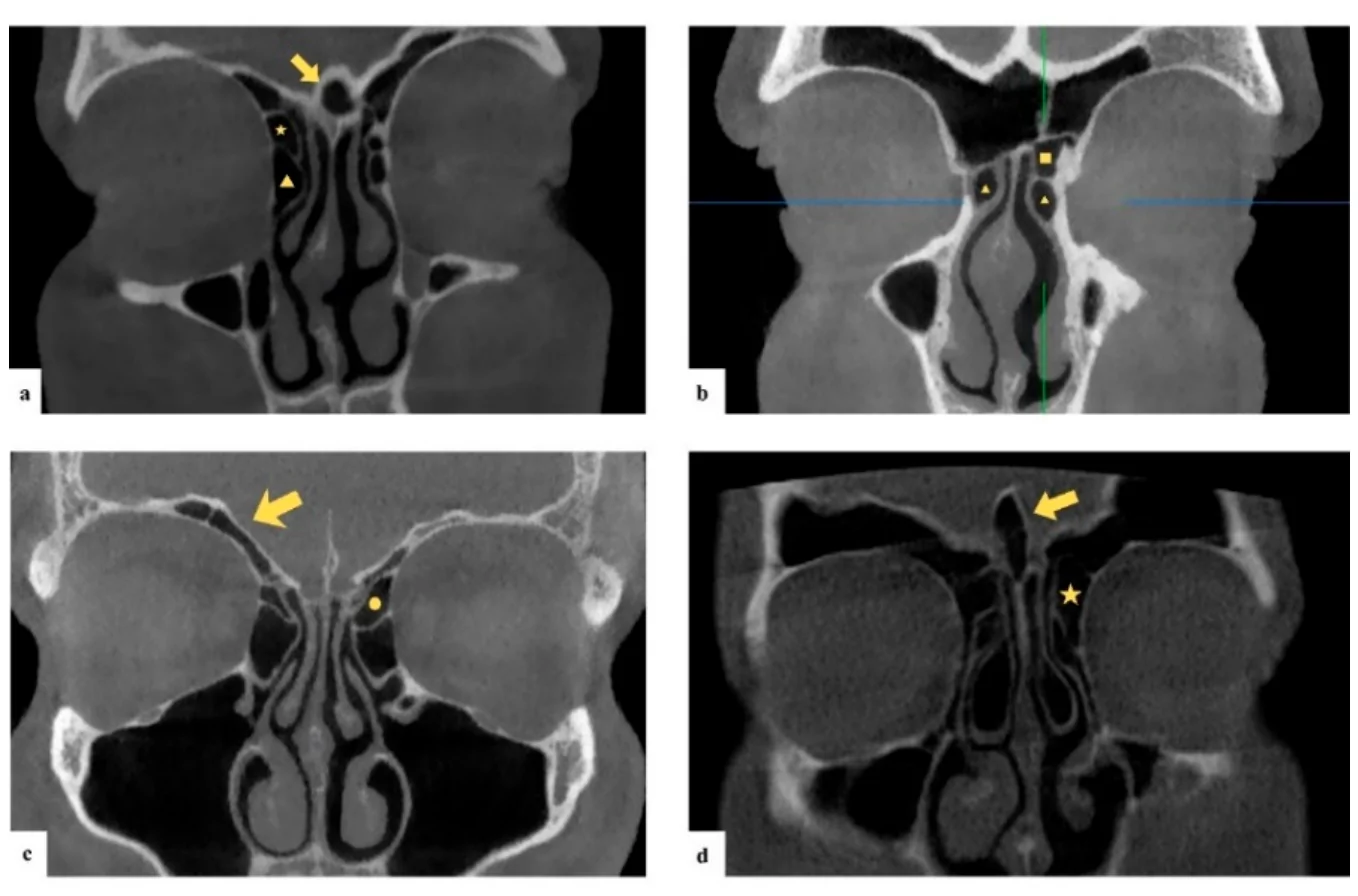
**Frontal sinus variants (IFAC). **(**a**) Coronal CBCT image showing a frontal septal cell (arrow), an agger nasi cell (triangle) and a supra-agger cell (star). (**b**) Coronal CBCT image demonstrating an agger nasi cell (triangle) and a supra-agger frontal cell (square). (The blue and green lines are own markings of the software of tomographic images.) (**c**) Coronal CBCT image illustrating a supraorbital ethmoidal cell (arrow) and a suprabullar cell (circle). (**d**) Coronal CBCT image demonstrating pneumatisation of the crista galli (arrow) and a supra-bulla frontal cell (star). IFAC, International Frontal Sinus Anatomy Classification.

Frontal sinus variations were classified according to the International Frontal Sinus Anatomy Classification (IFAC), following the groups described by Howser et al. [22]. Variants were grouped as follows (Figure 3):

- **Anterior:** Agger nasi cell, supra-agger cell and supra-agger frontal cell.
- **Posterior:** Supra-bulla cell, supra-bulla frontal cell and supraorbital ethmoid cell.
- **Medial:** Frontal septal cell.

### 2.6. Ethmoid Sinus Variations

**Figure 4:**
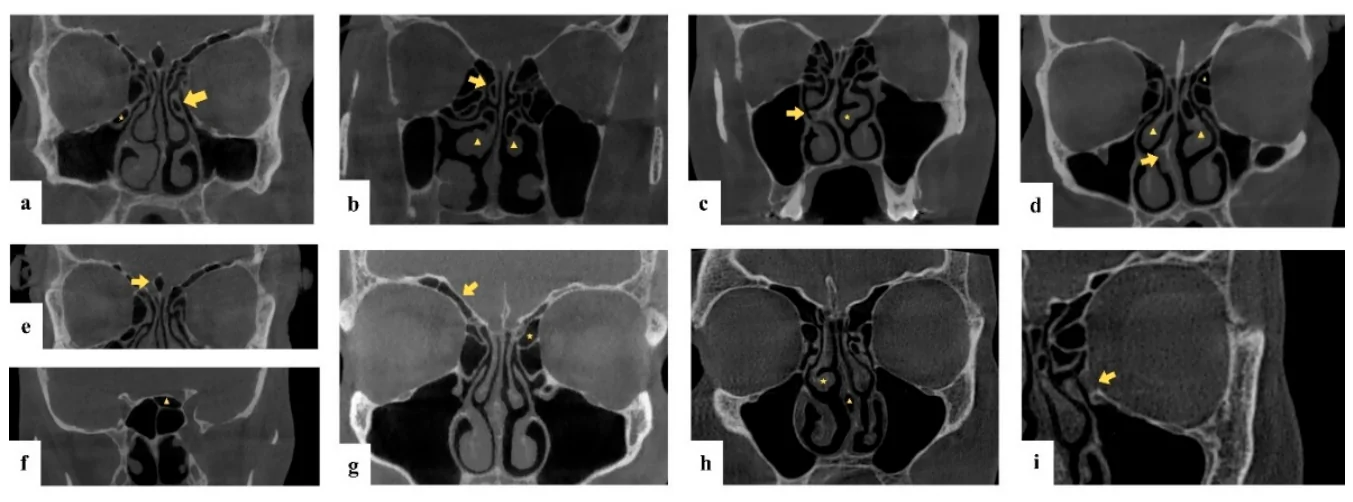
**Ethmoid sinus variations.** Coronal CBCT images showing ethmoid variations: (**a**) Haller cell (star) and pneumatised uncinate process (arrow); (**b**) pneumatised middle turbinate (triangle) and pneumatised superior turbinate (arrow); (**c**) septal spur (arrow) and paradoxical middle turbinate (star); (**d**) septal spur (arrow), pneumatised middle turbinate (triangle) and suprabullar cell (star); (**e**) pneumatised crista galli (arrow); (**f**) Onodi cell (triangle); (**g**) supraorbital ethmoidal cell (arrow) and suprabullar cell (star); (**h**) paradoxical middle turbinate (star) and septal pneumatisation (triangle); (**i**) focal dehiscence of the lamina papyracea (arrow).

Ethmoidal variations were classified into 13 categories based on the criteria described by Gibelli et al. [5]: septal pneumatisation, septal spur, pneumatised middle turbinate, paradoxical middle turbinate, pneumatised superior turbinate, pneumatised uncinate process, suprabullar cells, agger nasi cells, Onodi cells, Haller cells, pneumatised crista galli, supraorbital ethmoid cells and focal dehiscence of the lamina papyracea (Figure 4).

### 2.7. Sphenoid Sinus Variations

Sphenoid sinus morphology was evaluated in two dimensions using the classification proposed by Bilgir and Bayrakdar [7].

#### 2.7.1. Posteroanterior Pneumatisation

**Figure 5:**
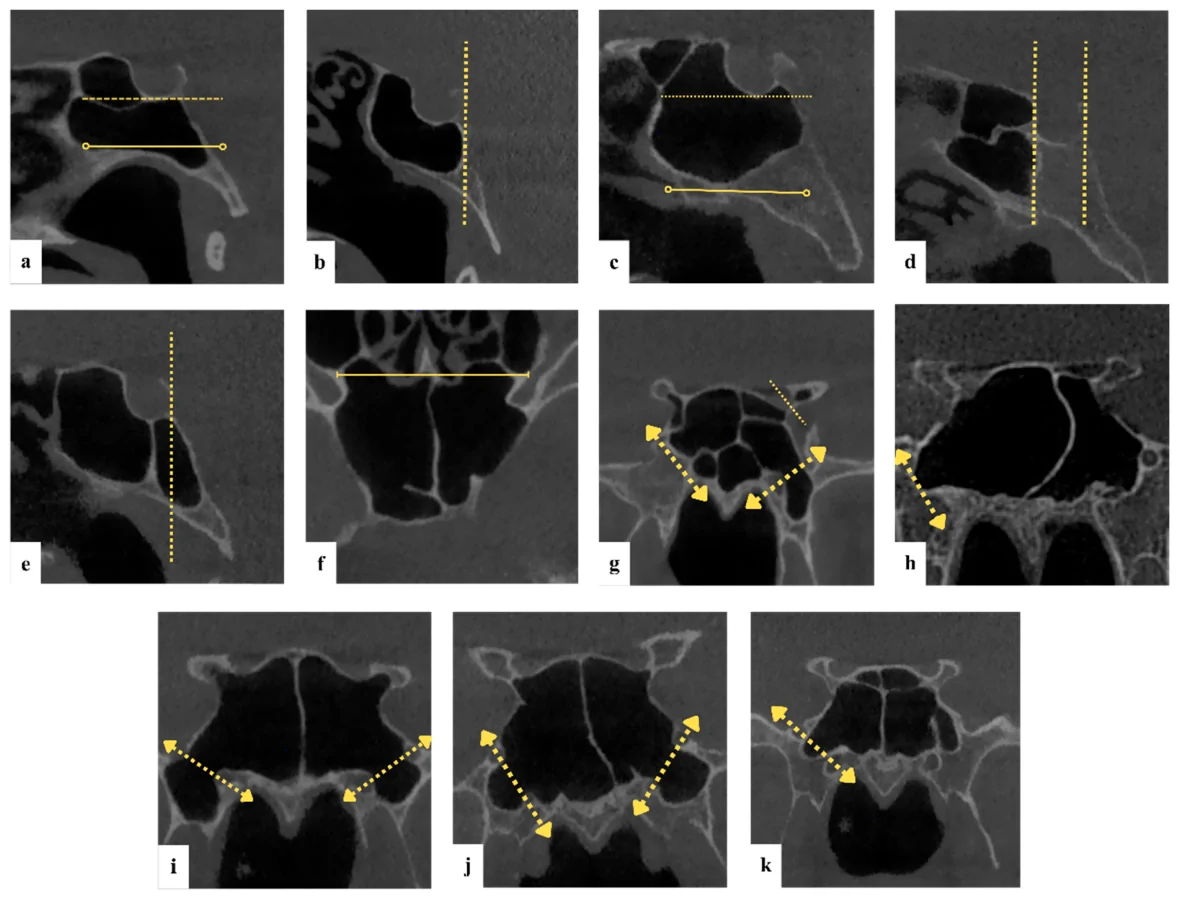
**Sphenoid sinus pneumatisation.** Sagittal CBCT images illustrating the posteroanterior classification of the sphenoid sinus: (**a**) clival type; (**b**) sellar type; (**c**) dorsum type; (**d**) presellar type; (**e**) combined anterior type with postsellar extension. (**f**) Axial CBCT image showing the combined anterior type with postsellar extension. Coronal CBCT images depicting the lateral classification of the sphenoid sinus: (**g**) lesser wing type on the right side and combined type on the left side; (**h**) lateral body type bilaterally; (**i**) inferior body–inferior type on the right side and greater wing–inferior type on the left side; (**j**) pterygoid inferior type; (**k**) lateral inferior type on the right side.

Five main types were defined: conchal, presellar, sellar, postsellar and anterior. The postsellar type was further subdivided into dorsum, subdorsum, occipital and full clival patterns. Classification was based on two horizontal reference planes passing through the floor of the hypophyseal fossa and the anterior opening of the vidian canal (Figure 5).

#### 2.7.2. Lateral Pneumatisation

Lateral extension was categorised into four groups: lateral body, lesser wing, inferior and combined. Reference landmarks included the optic canal, anterior clinoid process and the vidian canal–foramen rotundum line. The inferior type was further subdivided into inferior corpus, pterygoid inferior, greater wing inferior and full lateral pneumatisation (Figure 5).

### 2.8. Statistical Analysis

Continuous variables (age) are presented as mean ± standard deviation and median (range), and categorical variables as counts and percentages. Temporal pneumatisation (acellular < hypopneumatisation < moderate < good < hyperpneumatisation) and frontal sinus size (aplasia < hypoplasia < medium < hyperplasia) were analysed as ordinal variables; IFAC cells, frontal septal cells and ethmoid variants as binary variables; and the sphenoid lateral and posteroanterior (PA) types as nominal variables. For inferential analyses, sparse categories were merged (acellular with hypopneumatisation; aplasia with hypoplasia), and binary variants with fewer than five observations in either category (right supra-agger frontal cell, right supra-bulla frontal cell and septal pneumatisation) were reported descriptively only.

Bilateral concordance, defined as agreement between the right and left sides of the same individual, was quantified using unweighted Cohen’s κ for binary and nominal variables and quadratic-weighted κ for ordinal variables [23], with 95% confidence intervals (CIs) derived from 2000 non-parametric bootstrap resamples. κ values were interpreted according to Landis and Koch [24] as poor (<0.20), fair (0.21–0.40), moderate (0.41–0.60), good (0.61–0.80) and very good (>0.80). These indices describe anatomical symmetry and are not measures of observer reliability. Side asymmetry was tested using the exact McNemar test for binary variables and the Wilcoxon signed-rank test for ordinal variables.

Associations with age were tested using Spearman’s correlation for ordinal outcomes, the Mann–Whitney *U* test with Cohen’s *d* for binary outcomes and the Kruskal–Wallis *H* test with ε^2^ for nominal outcomes. Sex differences were tested using the Mann–Whitney *U* test with the rank-biserial correlation (*r*_rb_) for ordinal outcomes, Fisher’s exact test with the conditional maximum-likelihood odds ratio (OR) and exact 95% CI for binary outcomes and the Fisher–Freeman–Halton test (Monte Carlo *p* value, 20,000 replicates) with Cramér’s *V* for nominal outcomes. Effect sizes were interpreted using conventional thresholds [25]. Inter-regional associations were tested between variables belonging to different anatomical regions (temporal bone; frontal sinus, comprising sinus size, IFAC cells and frontal septal cells; ethmoid sinus; sphenoid sinus) using the test appropriate to each pair of variable types. Bilateral pairs, pairs within the same region and pairs in which the same air cell was recorded under both the IFAC and the ethmoid classification (agger nasi, supra-bulla/suprabullar and supraorbital ethmoidal cells) were excluded because they do not represent independent hypotheses. The association between the sphenoid lateral and PA types was analysed separately as a within-region comparison.

Multiple testing was controlled using the Benjamini–Hochberg false discovery rate (FDR) procedure [26], applied separately within four predefined test families: side asymmetry (6 tests), age (30 tests), sex (30 tests) and inter-regional associations (280 tests). The FDR approach was chosen because the analyses were exploratory and family-wise error control is overly conservative across many correlated tests; the Benjamini–Yekutieli procedure, which remains valid under arbitrary dependence between tests [27], was applied as a sensitivity analysis. Exact *p* values are reported. A two-sided FDR-adjusted *p* value < 0.05 was considered statistically significant; findings with a raw *p* value < 0.05 that did not survive FDR correction are reported as exploratory and are not interpreted as established associations.

Multivariable logistic regression was used in an exploratory manner to examine whether the strongest univariable signals persisted after adjustment. Age and sex were included in all models as a priori confounders, and additional predictors were selected from variables with a raw *p* value < 0.05 in the univariable analyses. Three models were fitted: (Model 1) frontal septal cell ∼ age + sex + ethmoid agger nasi cell; (Model 2) left temporal hyperpneumatisation ∼ age + sex + postsellar PA sphenoid type + left frontal hyperplasia; and (Model 3) right supraorbital ethmoidal (IFAC) cell ∼ age + sex. Because the number of outcome events was limited (events per parameter, 7.3–12.0) [28], Firth’s penalised-likelihood logistic regression [29] was used to reduce small-sample bias, and adjusted ORs are reported with 95% profile penalised-likelihood CIs.

To explore the multivariate structure of pneumatisation, 12 variables (right/left temporal pneumatisation, right/left frontal sinus size, right/left IFAC agger nasi, supra-bulla and supraorbital ethmoidal cells, pneumatised middle turbinate and Onodi cells) were analysed using multiple correspondence analysis (MCA), which is suited to categorical data [30]. The importance of each dimension was described by Benzécri-adjusted inertia, and the association of each variable with each dimension by the squared correlation ratio (η^2^). Because principal component analysis (PCA) assumes continuous variables, the suitability of the data for PCA was assessed using the Kaiser–Meyer–Olkin (KMO) measure [31] and Bartlett’s test of sphericity [32], computed on both Pearson and polychoric/tetrachoric correlation matrices.

Analyses were performed in R version 4.6.1 (R Foundation for Statistical Computing, Vienna, Austria) using the irr (version 0.85), psych (version 2.6.9), logistf (version 1.26.1) and FactoMineR (version 2.18) packages.

### 2.9. Use of Generative Artificial Intelligence

Generative artificial intelligence (AI) tools were used only for writing support. An open-weight large language model accessed through Ollama (https://ollama.com/; [accessed on 12 May 2026]; model v0.23.4d) was used to help present the statistical results in terms accessible to dentomaxillofacial radiologists.

## 3. Results

### 3.1. Demographics

The demographic characteristics of the study group are summarised in Table 1. The study group consisted of 88 patients (49 women, 55.7%; 39 men, 44.3%) with a mean age of 41.6 ± 13.3 years (median 38.5; range 25–75; 25th–75th percentile 30–51). There was no significant difference in age between the sexes (female 40.6 ± 13.4 vs. male 43.0 ± 13.1 years; Mann–Whitney *U* test, *p* = 0.293).

### 3.2. Prevalence of Pneumatisation Patterns

Temporal bone hyperpneumatisation was observed on 42.0% of right and 52.3% of left sides, and frontal sinus hyperplasia on 35.2% of right and 52.3% of left sides; side asymmetry is analysed in Section 3.3. One patient presented with right-sided absence of mastoid air cells; this pattern is not included in the existing classification and is reported here as an acellular variant. Frontal septal cells were present in 22 patients (25.0%). The distributions of temporal bone pneumatisation, frontal sinus size, frontal septal cells and sphenoid sinus pneumatisation are presented in Table 2, and those of IFAC and ethmoid variants in Appendix A (Table A1 and Table A2).

Among IFAC cells, agger nasi cells were the most common (right 68.2%, left 69.3%), followed by supra-bulla cells (right 37.5%, left 42.0%) and supraorbital ethmoidal cells (right 27.3%, left 35.2%). The most frequent ethmoid air cell variations were agger nasi cells (70.5%), pneumatised middle turbinate (58.0%), septal spur (55.7%), suprabullar cells (44.3%) and Onodi cells (40.9%). Focal dehiscence of the lamina papyracea was identified in 8.0% of the study group. Lateral pneumatisation of the sphenoid sinus was most commonly observed as the lateral body type (right side 42.0%, left side 34.1%). In the posteroanterior plane, the postsellar full clival subtype was the most frequent pattern (31.8%).

### 3.3. Bilateral Concordance and Side Asymmetry

All paired structures showed right–left concordance exceeding chance (Table 3). Concordance was good for supra-bulla (κ = 0.76, 95% CI 0.61–0.88), supraorbital ethmoidal (κ = 0.61, 95% CI 0.42–0.77) and agger nasi cells (κ = 0.60, 95% CI 0.41–0.77); moderate for temporal pneumatisation (weighted κ = 0.56, 95% CI 0.41–0.70), the sphenoid lateral type (κ = 0.53, 95% CI 0.39–0.67) and frontal sinus size (weighted κ = 0.42, 95% CI 0.24–0.58); and fair for supra-agger cells (κ = 0.32, 95% CI 0.07–0.56), whose wide CI is consistent with the low prevalence of this variant. Frontal sinus size was greater on the left side (left > right in 30 patients and right > left in eight; Wilcoxon signed-rank test, *p* < 0.001; FDR-adjusted *p* = 0.002). Temporal pneumatisation was more advanced on the left side in 27 patients and on the right side in 15 (*p* = 0.018), but this difference did not remain significant after FDR correction (*p* = 0.055). No side asymmetry was found for IFAC cells (all *p* ≥ 0.118).

### 3.4. Age- and Sex-Related Associations

Thirty outcomes were tested for associations with age and 30 for associations with sex (Table A3 and Table A4). At the uncorrected level, the septal spur was associated with younger age (38.0 ± 11.8 vs. 46.2 ± 13.7 years; Cohen’s *d* = −0.65; *p* = 0.005), and frontal septal cells (38.5% vs. 14.3%; OR = 3.69, 95% CI 1.21–12.31; *p* = 0.013), right supraorbital ethmoidal cells (41.0% vs. 16.3%; OR = 3.51, 95% CI 1.20–11.06; *p* = 0.015) and a larger left frontal sinus (*r*_rb_ = 0.26; *p* = 0.017) were more frequent in men, whereas Onodi cells were less frequent in men (28.2% vs. 51.0%; OR = 0.38, 95% CI 0.14–1.00; *p* = 0.049) (Table 4). None of these associations remained significant after FDR correction (all adjusted *p* ≥ 0.154); they are therefore regarded as exploratory.

### 3.5. Inter-Regional Associations

After the exclusion of bilateral, within-region and cross-classification (tautological) pairs, 280 inter-regional tests were performed. Fourteen (5.0%) reached *p* < 0.05 at the uncorrected level, a proportion close to that expected by chance, and none remained significant after FDR correction (all adjusted *p* ≥ 0.637; Table 5). Temporal pneumatisation showed only uncorrected associations with lamina papyracea dehiscence (*p* = 0.030) and the sphenoid PA type (*p* = 0.041). Within the sphenoid sinus, the left lateral type was associated with the PA type (Cramér’s *V* = 0.33, *p* < 0.001), whereas the right lateral type was not (*V* = 0.23, *p* = 0.133).

### 3.6. Exploratory Multivariable Logistic Regression

Three exploratory multivariable logistic regression models were fitted using Firth’s penalised likelihood (Table 6). In Model 1, male sex remained associated with frontal septal cells after adjustment for age and ethmoid agger nasi cells (adjusted OR = 3.75, 95% CI 1.36–11.15; *p* = 0.010), and ethmoid agger nasi cells were also associated with frontal septal cells (adjusted OR = 4.81, 95% CI 1.31–26.29; *p* = 0.016). In Model 2, none of the predictors was associated with left temporal hyperpneumatisation. In Model 3, male sex was associated with right supraorbital ethmoidal cells after adjustment for age (adjusted OR = 3.48, 95% CI 1.35–9.62; *p* = 0.010).

Because the predictors were selected on the basis of univariable screening and the corresponding univariable associations did not survive FDR correction, these adjusted associations are regarded as hypothesis-generating. The wide CIs, particularly for ethmoid agger nasi cells, reflect the limited number of outcome events.

### 3.7. Multiple Correspondence Analysis

**Figure 6:**
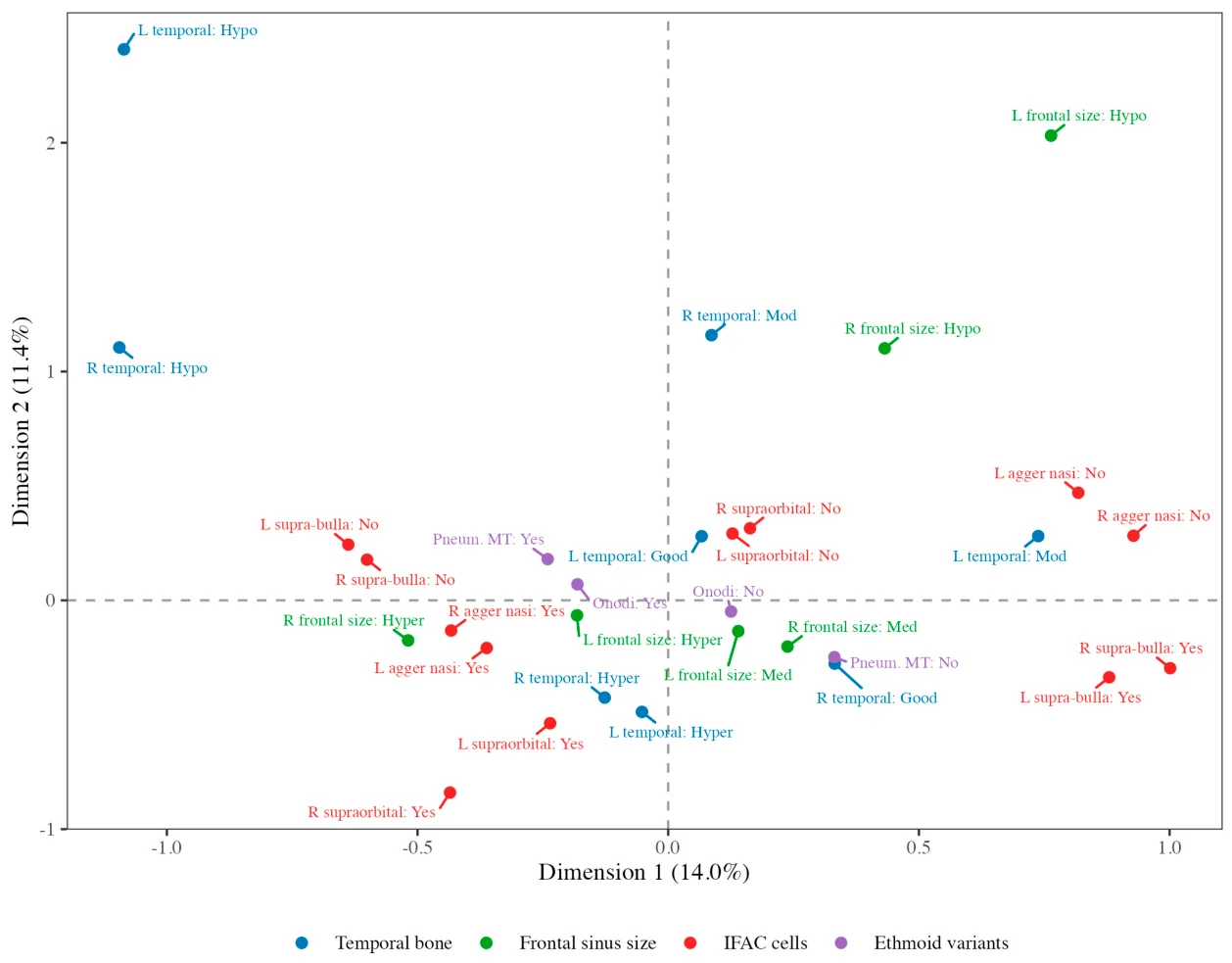
Multiple correspondence analysis (MCA) map of the categories of the 12 pneumatisation variables on the first two dimensions (*n* = 88). Dimension 1 contrasts the presence of IFAC supra-bulla cells with the presence of agger nasi cells, whereas dimension 2 is dominated by temporal pneumatisation. Hypo, hypopneumatisation/hypoplasia; Mod, moderate; Med, medium; Hyper, hyperpneumatisation/hyperplasia; IFAC, International Frontal Sinus Anatomy Classification; L, left; MT, middle turbinate; Pneum., pneumatised; R, right.

The data were not suitable for PCA: although Bartlett’s test was significant (χ^2^ = 282.9, *p* < 0.001), the KMO measure was 0.45 and did not improve when computed on the polychoric/tetrachoric correlation matrix (0.13; the matrix was not positive definite). The 12 variables were therefore analysed using MCA. The first three dimensions accounted for 14.0%, 11.4% and 9.6% of the total inertia (52.7%, 25.3% and 12.2% after Benzécri adjustment; Table 7). Dimension 1 was defined mainly by IFAC supra-bulla cells (η^2^ = 0.60 on the right and 0.56 on the left) and agger nasi cells (η^2^ = 0.40 and 0.30), which were located on opposite sides of the axis; dimension 2 was defined mainly by temporal pneumatisation (η^2^ = 0.41 and 0.49), with smaller contributions from frontal sinus size and supraorbital ethmoidal cells; and dimension 3 by supraorbital ethmoidal cells and temporal pneumatisation (Figure 6; Table A5).

No single dimension captured the variability of all four regions, and frontal recess cells and temporal pneumatisation were represented on separate dimensions.

## 4. Discussion

This study evaluated temporal bone pneumatisation together with frontal, ethmoid and sphenoid sinus variations in the same individuals using CBCT. Because the design was retrospective and cross-sectional, the results describe associations rather than causal relationships and should be interpreted as exploratory. The most consistent findings were the moderate-to-good bilateral concordance of the evaluated structures and the left-sided predominance of frontal sinus size. In contrast, after the exclusion of tautological comparisons and correction for multiple testing, no association between anatomical regions remained statistically significant; at the uncorrected level, temporal bone pneumatisation was associated only with lamina papyracea dehiscence and the sphenoid posteroanterior type. Previous studies have reported such inter-regional relationships. Gibelli et al. [3] described significant correlations between temporal bone pneumatisation and ethmoidal and sphenoidal variants, particularly involving the petrous apex and infralabyrinthine compartments, and Hindi et al. [1] demonstrated parallel pneumatisation patterns of the mastoid and sphenoid sinuses. Similarly, Gulić et al. found that CT-measured mastoid volume correlated positively with sphenoid sinus volume [33]. These studies used region-specific or volumetric assessments, whereas categorical classifications were used here; together with the sample size, this may have limited the ability of the present study to detect weaker inter-regional associations, and the absence of significant findings should not be interpreted as evidence that such associations do not exist.

The left-sided predominance of frontal sinus size, the only finding that remained significant after correction for multiple testing, agrees with earlier reports. In a radiographic study of 469 adults, Kanat et al. found the left frontal sinus to be dominant in 50.1% and the right in 35.6% of cases [34]. Using CT-based volumetry and the same four size categories as the present study, Yüksel Aslier et al. observed hyperplasia more often on the left side and hypoplasia more often on the right [35]. In a CBCT study of a South Indian population, Denny et al. found the left frontal sinus to be larger in most dimensions, although only the anteroposterior dimension in men differed significantly [36]. Kanat et al. suggested that cerebral hemispheric dominance and handedness may contribute to this asymmetry [34]; handedness was not recorded in the present study, so this explanation could not be tested.

The frequencies of individual variants in the present sample differed in part from previous reports. In a CT study of 509 Turkish adults, Yaylacı and Değer found agger nasi cells in 88.0%, supra-bulla cells in 43.0%, frontal septal cells in 22.0% and supraorbital ethmoid cells in 17.1% of cases [37], and Braz et al. reported agger nasi cells in 95.6% of a Brazilian population [38]. Frontal septal and supra-bulla cell frequencies in the present study (25.0% and 37.5–42.0%) were close to these figures, whereas agger nasi cells were less frequent (68.2–69.3%) and supraorbital ethmoidal cells more frequent (27.3–35.2%). For the sphenoid sinus, Tavakoli et al. found a postsellar type in 82.5% and Onodi cells in 28.9% of 97 CBCT scans [39], compared with 47.7% and 40.9% in the present study. These differences may reflect population characteristics, differences between CBCT and multidetector CT and differences in how individual cells were defined, so frequencies obtained with different imaging methods should be compared with caution.

One patient presented with an acellular mastoid process, a pattern not defined within the reference classification system used for temporal bone pneumatisation [19,20]. Consequently, this case was classified as acellular. This finding highlights the presence of a pneumatisation variant that is not currently included in literature-based classifications and may indicate a limitation of existing systems or population-related variation in temporal bone anatomy. This pattern may represent a rare anatomical type. The absence of complete patient anamnesis did not allow the exclusion of asymptomatic aetiological factors.

The sex-related differences observed are consistent with findings reported in the literature. The umbrella review by Anees et al. [8] highlighted consistent sexual dimorphism across the frontal, maxillary and sphenoid sinuses. The present findings are in line with previous studies demonstrating a higher prevalence of frontal septal cells in males. Anatomical variations of the paranasal sinuses have been shown to contribute to forensic identification, particularly for sex determination. However, the forensic value of sphenoid sinus morphology remains sparsely explored, and sexual dimorphism of ethmoidal cells and sinuses has not yet been adequately addressed in the literature [8]. Volumetric analyses by Dhakal et al. [40], as well as studies by Benghiac et al. [41] and Chatra et al. [42], reported larger frontal sinus volumes in males. In the present study, frontal septal cells and right supraorbital ethmoidal cells were more frequent in men, and these differences persisted after adjustment for age in exploratory Firth regression (adjusted OR = 3.75 and 3.48, respectively). However, the corresponding univariable associations did not survive FDR correction; these findings should therefore be regarded as hypothesis-generating and require confirmation in larger independent samples.

The most comprehensive and contemporary classification of the sphenoid sinus was proposed by Bilgir and Bayrakdar [7] and was preferred in the present study because of the shared ethnic background of the study populations. Within this classification, postsellar and clival types are reported to exhibit greater lateral pneumatisation and a higher prevalence of Onodi cells. In the present cohort, the left lateral type was associated with the posteroanterior type, whereas no significant association was observed between the posteroanterior type and Onodi cells. The anatomical relationships among the sphenoid sinus, Onodi cells, internal carotid artery and optic nerve render these variations particularly significant from a surgical perspective [43]. Although a relationship between sphenoid and temporal pneumatisation has been reported [1,14], the association between the sphenoid posteroanterior type and temporal pneumatisation observed here at the uncorrected level did not survive correction for multiple testing.

MCA did not identify a single dimension capturing the variability of all regions: the first dimension was defined mainly by frontal recess cells (supra-bulla and agger nasi cells) [5,22], and the second by temporal pneumatisation. Regional analyses have occasionally reported correlations that do not generalise to other anatomical regions, and this decomposition may help to explain inconsistencies in the literature. In Anatolian skulls from different periods, Yücel et al. observed that temporal bone pneumatisation decreased over the centuries whereas paranasal sinus volumes did not change [44], which is consistent with the absence of a dimension common to temporal and paranasal pneumatisation in the present data. However, these dimensions describe patterns of statistical covariation in a cross-sectional sample and cannot establish developmental mechanisms; explanations based on transmitted pressure or independent developmental processes remain hypotheses that require longitudinal data.

Although disease susceptibility, surgical outcomes and forensic applications were not investigated in the present study, the variants evaluated here are of clinical interest because previous studies have suggested that they may influence patterns of pathological spread. Previous studies have suggested that sinus pneumatisation patterns and their variations may be involved in the initiation, persistence and dissemination of disease between adjacent compartments. Aeromedical studies have reported that the narrow and anatomically variable outflow tract of the frontal sinus may predispose this region to impaired ventilation and barometric pressure imbalance, even in the presence of minimal mucosal oedema, which may facilitate barotrauma and recurrent inflammation [45,46,47]. In line with this vulnerability, Shabto et al. [11] demonstrated that frontal sinus anatomical variants may be associated with unpredictable pathways of pathological spread between the frontal sinus and orbit, including intraosseous venous malformations, underscoring the clinical importance of frontal recess and ethmoid cell anatomy.

The studies by Patel and Govindaraj [48] and Sethi et al. [13] further suggest that variations of the ethmoid and sphenoid sinuses, and their anatomical interrelationships, extend beyond surgical relevance alone. Together, these studies suggest that sphenoethmoidal anatomy may be relevant not only to operative risk but also to the dissemination of disease.

This study has several strengths, including the evaluation of four anatomical regions in the same individuals, the use of established radio-morphological classifications, the inclusion of patients aged 25 years and older to ensure completion of anatomical development, the exclusion of tautological comparisons, FDR control within predefined test families [26], the reporting of exact *p* values and effect sizes with CIs and the use of statistical methods appropriate for sparse and categorical data.

Several limitations should also be acknowledged. First, the retrospective cross-sectional design precludes inferences about developmental mechanisms or causality. Second, the sample size limited the precision of the estimates, and several variants were rare, resulting in wide CIs; non-significant results should not be interpreted as evidence of no association. Third, patients were drawn from a single institutional archive of individuals referred for CBCT, which may introduce selection bias and limit generalisability to other populations. In addition, because the archive was screened consecutively until the required sample size was reached, the total number of screened records was not recorded. Fourth images were acquired with two CBCT systems using device-specific protocols, which may have affected the visualisation of small air cells. Fifth, categorical classifications rather than volumetric measurements were used, which reduces sensitivity to subtle differences in pneumatisation. Sixth, the otological history of the patients was not available; because middle ear disease has been associated with reduced mastoid pneumatisation without corresponding changes in the paranasal sinuses [49], this may have affected the temporal bone findings. Finally, the multivariable models were data-driven and should be validated in independent samples. Larger, multicentre and prospective studies including volumetric measurements and comparisons between normal and pathological cases are needed to clarify inter-regional relationships and their clinical implications.

## 5. Conclusions

Within the limits of this exploratory, retrospective cross-sectional study, the main findings were as follows:

- Temporal bone pneumatisation, frontal sinus size and frontal recess cells showed moderate-to-good bilateral concordance, and the frontal sinus was larger on the left side.
- After correction for multiple testing, no association between temporal bone pneumatisation and frontal, ethmoid or sphenoid variants was identified, and multivariate analysis did not reveal a single dimension common to all regions.
- Male sex was associated with frontal septal cells and right supraorbital ethmoidal cells in exploratory multivariable analyses; these findings are hypothesis-generating.
- Knowledge of the frequency and bilateral distribution of these variants may support radiological interpretation; larger sample sizes with multiple centres are needed to clarify inter-regional relationships.

## Figures and Tables

**Table 1:** Demographic characteristics of the study group (*n* = 88).

Characteristic	Value
Total, *n*	88
Women, *n* (%)	49 (55.7)
Men, *n* (%)	39 (44.3)
Age, mean ± SD (years)	41.6 ± 13.3
Age, median (range) (years)	38.5 (25–75)
Age, 25th–75th percentile (years)	30–51
Women, age, mean ± SD (years)	40.6 ± 13.4
Men, age, mean ± SD (years)	43.0 ± 13.1
Sex difference in age	*p* = 0.293 (Mann–Whitney *U* test)

SD, standard deviation.

**Table 2:** Distribution of temporal bone pneumatisation, frontal sinus size, frontal septal cells and sphenoid sinus pneumatisation (*n* = 88).

Variable	Category	Right, *n* (%)	Left, *n* (%)	Midline, *n* (%)
Temporal pneumatisation	Hypopneumatisation	5 (5.7)	5 (5.7)	–
	Moderate	15 (17.0)	8 (9.1)	–
	Good	30 (34.1)	29 (33.0)	–
	Hyperpneumatisation	37 (42.0)	46 (52.3)	–
	Acellular	1 (1.1)	0 (0.0)	–
Frontal sinus size	Aplasia	1 (1.1)	0 (0.0)	–
	Hypoplasia	12 (13.6)	4 (4.5)	–
	Medium	44 (50.0)	38 (43.2)	–
	Hyperplasia	31 (35.2)	46 (52.3)	–
Frontal septal cell	Present	–	–	22 (25.0)
	Absent	–	–	66 (75.0)
Sphenoid lateral type	Lateral body	37 (42.0)	30 (34.1)	**–**
	Lesser wing	18 (20.5)	13 (14.8)	–
	Inferior	21 (23.9)	27 (30.7)	–
	Combined	11 (12.5)	17 (19.3)	–
	Other ^a^	1 (1.1)	1 (1.1)	–
Sphenoid posteroanterior type	Conchal	–	–	1 (1.1)
	Presellar	–	–	15 (17.0)
	Sellar	–	–	22 (25.0)
	Postsellar	–	–	42 (47.7)
	Anterior	–	–	8 (9.1)

Percentages are based on *n* = 88. Frontal septal cells and the sphenoid posteroanterior type are midline assessments. ^a^ One patient had a bilateral sphenoid lateral pattern that could not be assigned to the four lateral groups.

**Table 3:** Bilateral (right–left) concordance and side asymmetry of temporal bone and paranasal sinus structures (*n* = 88).

Structure	Statistic	κ (95% CI)	Interpretation	Right/Left	*p* (Asymmetry)	FDR *p*
Temporal pneumatisation	Weighted κ	0.56 (0.41–0.70)	Moderate	3/4	0.018	0.055
Frontal sinus size	Weighted κ	0.42 (0.24–0.58)	Moderate	3/4	<0.001	0.002
Sphenoid lateral type	Cohen’s κ	0.53 (0.39–0.67)	Moderate	–	–	–
IFAC agger nasi	Cohen’s κ	0.60 (0.41–0.77)	Good	68.2%/69.3%	1.000	1.000
IFAC supra-agger	Cohen’s κ	0.32 (0.07–0.56)	Fair	18.2%/13.6%	0.454	0.545
IFAC supra-bulla	Cohen’s κ	0.76 (0.61–0.88)	Good	37.5%/42.0%	0.344	0.516
IFAC supraorbital ethmoidal	Cohen’s κ	0.61 (0.42–0.77)	Good	27.3%/35.2%	0.118	0.237

κ, Cohen’s kappa (binary and nominal variables); weighted κ, quadratic-weighted kappa (ordinal variables); 95% CIs from 2000 bootstrap resamples; interpretation according to Landis and Koch [24]. Right/left shows prevalence (binary variables) or median grade (ordinal variables; temporal: 0 = acellular to 4 = hyperpneumatisation; frontal: 1 = aplasia to 4 = hyperplasia). Side asymmetry: exact McNemar test (binary) or Wilcoxon signed-rank test (ordinal); FDR, Benjamini–Hochberg-adjusted *p* value within the asymmetry family (6 tests). These indices quantify anatomical symmetry and are not measures of observer reliability. CI, confidence interval; FDR, false discovery rate; IFAC, International Frontal Sinus Anatomy Classification.

**Table 4:** Age- and sex-related associations with a raw *p* value < 0.05 (*n* = 88).

Factor	Outcome	Test	Effect Size (95% CI)	*p*	BH	BY
Age	Ethmoid septal spur	Mann–Whitney *U*	*d* = −0.65 (present 38.0 ± 11.8 vs. absent 46.2 ± 13.7)	0.005	0.154	0.614
Sex	Frontal septal cell	Fisher exact	OR = 3.69 (1.21–12.31); M 38.5% vs. F 14.3%	0.013	0.165	0.660
	R IFAC supraorbital ethmoidal	Fisher exact	OR = 3.51 (1.20–11.06); M 41.0% vs. F 16.3%	0.015	0.165	0.660
	L frontal sinus size	Mann–Whitney *U*	*r*_rb_ = 0.26 (M vs. F)	0.017	0.165	0.660
	Ethmoid Onodi cell	Fisher exact	OR = 0.38 (0.14–1.00); M 28.2% vs. F 51.0%	0.049	0.366	1.000

Age family, 30 tests; sex family, 30 tests (complete results in Table A3 and Table A4). Effect sizes: Cohen’s *d* (variant present vs. absent); OR, conditional maximum-likelihood odds ratio with exact 95% CI (men vs. women); *r*_rb_, rank-biserial correlation (positive values indicate larger values in men). *p*, raw *p* value; BH, Benjamini–Hochberg FDR-adjusted *p* value; BY, Benjamini–Yekutieli FDR-adjusted *p* value. No association reached an FDR-adjusted *p* < 0.05. CI, confidence interval; F, female; FDR, false discovery rate; IFAC, International Frontal Sinus Anatomy Classification; L, left; M, male; OR, odds ratio; R, right.

**Table 5:** Inter-regional associations with a raw *p* value < 0.05 (280 tests; none significant after FDR correction; *n* = 88).

Variable 1	Variable 2	Test	Effect Size (95% CI)	*p*	BH
R IFAC supra-agger	Ethmoid agger nasi cell	Fisher exact	OR = ∞ (1.95–∞)	0.002	0.637
R IFAC supra-bulla	L sphenoid lateral type	Fisher–Freeman–Halton	*V* = 0.37	0.012	0.839
Frontal septal cell	Ethmoid agger nasi cell	Fisher exact	OR = 5.62 (1.19–53.79)	0.016	0.839
R IFAC supra-bulla	Ethmoid agger nasi cell	Fisher exact	OR = 0.30 (0.10–0.86)	0.016	0.839
L IFAC supra-bulla	Ethmoid septal spur	Fisher exact	OR = 0.35 (0.13–0.89)	0.018	0.839
Ethmoid agger nasi cell	L sphenoid lateral type	Fisher–Freeman–Halton	*V* = 0.37	0.024	0.839
R IFAC supra-bulla	Ethmoid pneumatised middle turbinate	Fisher exact	OR = 0.36 (0.13–0.96)	0.027	0.839
R IFAC agger nasi	Ethmoid pneumatised superior turbinate	Fisher exact	OR = 5.06 (1.06–48.73)	0.027	0.839
Ethmoid septal spur	L sphenoid lateral type	Fisher–Freeman–Halton	*V* = 0.34	0.028	0.839
L temporal pneumatisation	Ethmoid lamina papyracea dehiscence	Mann–Whitney *U*	*r*_rb_ = −0.45	0.030	0.839
R frontal sinus size	Ethmoid pneumatised crista galli	Mann–Whitney *U*	*r*_rb_ = 0.51	0.038	0.864
R IFAC agger nasi	Ethmoid suprabullar cell	Fisher exact	OR = 0.38 (0.13–1.04)	0.041	0.864
R temporal pneumatisation	Sphenoid posteroanterior type	Kruskal–Wallis *H*	ε^2^ = 0.11	0.041	0.864
L IFAC agger nasi	Ethmoid pneumatised superior turbinate	Fisher exact	OR = 4.76 (1.00–45.88)	0.047	0.864

Only the 14 of 280 inter-regional tests with a raw *p* < 0.05 are shown; none remained significant after FDR correction. Fisher exact: conditional maximum-likelihood OR with exact 95% CI; Fisher–Freeman–Halton: Monte Carlo *p* value (20,000 replicates) with Cramér’s *V*; Mann–Whitney *U*: rank-biserial correlation (*r*_rb_); Kruskal–Wallis *H*: ε^2^. By definition, a supra-agger cell lies above an agger nasi cell, which explains the infinite OR in the first row. BH, Benjamini–Hochberg FDR-adjusted *p* value; CI, confidence interval; FDR, false discovery rate; IFAC, International Frontal Sinus Anatomy Classification; L, left; OR, odds ratio; PA, posteroanterior; R, right.

**Table 6:** Exploratory multivariable logistic regression models (Firth penalised likelihood; *n* = 88).

Model/Outcome	Predictor	Adjusted OR (95% CI)	*p*
Model 1: frontal septal cell (events = 22; EPP = 7.3)	Age (per year)	1.00 (0.96–1.04)	0.849
	Male sex	3.75 (1.36–11.15)	0.010
	Ethmoid agger nasi cell	4.81 (1.31–26.29)	0.016
Model 2: L temporal hyperpneumatisation (events = 46; EPP = 10.5)	Age (per year)	1.02 (0.99–1.06)	0.132
	Male sex	0.96 (0.40–2.30)	0.918
	Postsellar PA sphenoid type	0.61 (0.26–1.39)	0.239
	L frontal hyperplasia	0.80 (0.34–1.91)	0.621
Model 3: R supraorbital ethmoidal IFAC cell (events = 24; EPP = 12.0)	Age (per year)	0.99 (0.95–1.03)	0.578
	Male sex	3.48 (1.35–9.62)	0.010

Firth penalised-likelihood logistic regression with 95% profile penalised-likelihood CIs and penalised likelihood-ratio *p* values. Reference categories: female sex and absence of the predictor variant. EPP, events per parameter (number of observations in the smaller outcome category divided by the number of predictors). CI, confidence interval; IFAC, International Frontal Sinus Anatomy Classification; L, left; OR, odds ratio; PA, posteroanterior; R, right.

**Table 7:** Inertia explained by the first five dimensions of the multiple correspondence analysis (*n* = 88).

Dimension	Eigenvalue	Inertia (%)	Benzécri-Adjusted Inertia (%)
1	0.209	14.0	52.7
2	0.171	11.4	25.3
3	0.144	9.6	12.2
4	0.126	8.4	6.1
5	0.116	7.8	3.6

Benzécri adjustment was applied to eigenvalues > 1/*Q* (*Q* = 12 variables). Variable–dimension η^2^ values are given in Table A5. MCA, multiple correspondence analysis.

## Data Availability

The data presented in this study are not publicly available and cannot be shared because of patient privacy and ethical restrictions.

## Abbreviations

The following abbreviations are used in this manuscript:

CBCT	Cone-beam computed tomography
CI	Confidence interval
EPP	Events per parameter
FDR	False discovery rate
IFAC	International Frontal Sinus Anatomy Classification
KMO	Kaiser–Meyer–Olkin
MCA	Multiple correspondence analysis
MOL	Midorbital line
MPR	Multiplanar reconstruction
OR	Odds ratio
PA	Posteroanterior
PCA	Principal component analysis
SD	Standard deviation
SOL	Supraorbital line
SS	Sigmoid sinus

## Appendix A

The following tables present the full underlying data summarised in the main text.

**Table A1:** Distribution of IFAC frontal recess variants (*n* = 88).

IFAC.	Right, *n* (%)	Left, *n* (%)
Agger nasi	60 (68.2)	61 (69.3)
Supra-agger	16 (18.2)	12 (13.6)
Supra-agger frontal	2 (2.3)	5 (5.7)
Supra-bulla	33 (37.5)	37 (42.0)
Supra-bulla frontal	2 (2.3)	5 (5.7)
Supraorbital ethmoidal	24 (27.3)	31 (35.2)
No anterior cell visible	7 (8.0)	7 (8.0)

IFAC, International Frontal Sinus Anatomy Classification.

**Table A2:** Distribution of ethmoid (Gibelli) and sphenoid (Bilgir–Bayrakdar) variants (*n* = 88).

Variant/Pattern	*n* (%)
Ethmoid: agger nasi cells	62 (70.5)
Ethmoid: pneumatised middle turbinate	51 (58.0)
Ethmoid: septal spur	49 (55.7)
Ethmoid: suprabullar cells	39 (44.3)
Ethmoid: Onodi cells	36 (40.9)
Ethmoid: supraorbital ethmoid cells	34 (38.6)
Ethmoid: Haller cells	29 (33.0)
Ethmoid: pneumatised superior turbinate	19 (21.6)
Ethmoid: paradoxical middle turbinate	13 (14.8)
Ethmoid: pneumatised uncinate process	9 (10.2)
Ethmoid: focal dehiscence of lamina papyracea	7 (8.0)
Ethmoid: pneumatised crista galli	5 (5.7)
Ethmoid: septal pneumatisation	2 (2.3)
Sphenoid lateral (R): lateral body	37 (42.0)
Sphenoid lateral (L): lateral body	30 (34.1)
PA sphenoid: postsellar full clival (4d)	28 (31.8)
PA sphenoid: sellar (3)	22 (25.0)
PA sphenoid: presellar (2)	15 (17.0)
PA sphenoid: postsellar dorsum (4b)	14 (15.9)

L, left; PA, posteroanterior; R, right.

**Table A3:** Age-related associations: all 30 tests (*n* = 88).

Outcome	Test	Effect Size	*p*	BH	BY
R temporal pneumatisation	Spearman	ρ = 0.16	0.144	0.619	1.000
L temporal pneumatisation	Spearman	ρ = 0.12	0.267	0.890	1.000
R frontal sinus size	Spearman	ρ = 0.18	0.094	0.565	1.000
L frontal sinus size	Spearman	ρ = −0.01	0.921	0.995	1.000
R IFAC agger nasi	Mann–Whitney *U*	*d* = −0.31 (present 40.3 ± 12.8 vs. absent 44.5 ± 14.1)	0.191	0.715	1.000
R IFAC supra-agger	Mann–Whitney *U*	*d* = 0.00 (present 41.7 ± 14.1 vs. absent 41.6 ± 13.2)	0.974	0.995	1.000
R IFAC supra-bulla	Mann–Whitney *U*	*d* = 0.06 (present 42.1 ± 12.3 vs. absent 41.4 ± 13.9)	0.714	0.995	1.000
R IFAC supraorbital ethmoidal	Mann–Whitney *U*	*d* = −0.08 (present 40.9 ± 9.8 vs. absent 41.9 ± 14.4)	0.866	0.995	1.000
L IFAC agger nasi	Mann–Whitney *U*	*d* = −0.47 (present 39.8 ± 11.6 vs. absent 45.9 ± 15.9)	0.113	0.565	1.000
L IFAC supra-agger	Mann–Whitney *U*	*d* = 0.10 (present 42.8 ± 14.1 vs. absent 41.5 ± 13.2)	0.618	0.995	1.000
L IFAC supra-agger frontal	Mann–Whitney *U*	*d* = −0.10 (present 40.4 ± 17.1 vs. absent 41.7 ± 13.1)	0.685	0.995	1.000
L IFAC supra-bulla	Mann–Whitney *U*	*d* = 0.07 (present 42.2 ± 12.3 vs. absent 41.2 ± 14.1)	0.594	0.995	1.000
L IFAC supra-bulla frontal	Mann–Whitney *U*	*d* = −0.00 (present 41.6 ± 18.1 vs. absent 41.7 ± 13.1)	0.843	0.995	1.000
L IFAC supraorbital ethmoidal	Mann–Whitney *U*	*d* = −0.04 (present 41.3 ± 11.5 vs. absent 41.8 ± 14.3)	0.820	0.995	1.000
Frontal septal cell	Mann–Whitney *U*	*d* = −0.10 (present 40.6 ± 11.7 vs. absent 42.0 ± 13.8)	0.750	0.995	1.000
Ethmoid septal spur	Mann–Whitney *U*	*d* = −0.65 (present 38.0 ± 11.8 vs. absent 46.2 ± 13.7)	0.005	0.154	0.614
Ethmoid pneumatised middle turbinate	Mann–Whitney *U*	*d* = −0.20 (present 40.5 ± 12.9 vs. absent 43.2 ± 13.8)	0.419	0.995	1.000
Ethmoid paradoxical middle turbinate	Mann–Whitney *U*	*d* = 0.04 (present 42.1 ± 14.3 vs. absent 41.6 ± 13.2)	0.995	0.995	1.000
Ethmoid pneumatised superior turbinate	Mann–Whitney *U*	*d* = 0.53 (present 47.1 ± 15.5 vs. absent 40.2 ± 12.3)	0.089	0.565	1.000
Ethmoid pneumatised uncinate process	Mann–Whitney *U*	*d* = −0.21 (present 39.1 ± 12.1 vs. absent 41.9 ± 13.5)	0.469	0.995	1.000
Ethmoid suprabullar cell	Mann–Whitney *U*	*d* = −0.07 (present 41.1 ± 11.9 vs. absent 42.1 ± 14.4)	0.926	0.995	1.000
Ethmoid agger nasi cell	Mann–Whitney *U*	*d* = −0.42 (present 40.0 ± 12.5 vs. absent 45.5 ± 14.5)	0.110	0.565	1.000
Ethmoid Onodi cell	Mann–Whitney *U*	*d* = −0.06 (present 41.2 ± 13.2 vs. absent 42.0 ± 13.5)	0.809	0.995	1.000
Ethmoid Haller cell	Mann–Whitney *U*	*d* = 0.43 (present 45.4 ± 14.5 vs. absent 39.8 ± 12.3)	0.105	0.565	1.000
Ethmoid pneumatised crista galli	Mann–Whitney *U*	*d* = −0.08 (present 40.6 ± 12.6 vs. absent 41.7 ± 13.4)	0.971	0.995	1.000
Ethmoid supraorbital ethmoid cell	Mann–Whitney *U*	*d* = −0.15 (present 40.4 ± 11.5 vs. absent 42.4 ± 14.3)	0.709	0.995	1.000
Ethmoid lamina papyracea dehiscence	Mann–Whitney *U*	*d* = −0.22 (present 39.0 ± 7.5 vs. absent 41.9 ± 13.7)	0.829	0.995	1.000
R sphenoid lateral type	Kruskal–Wallis *H*	ε^2^ = 0.04	0.538	0.995	1.000
L sphenoid lateral type	Kruskal–Wallis *H*	ε^2^ = 0.03	0.576	0.995	1.000
Sphenoid posteroanterior type	Kruskal–Wallis *H*	ε^2^ = 0.03	0.701	0.995	1.000

Spearman: Spearman’s correlation coefficient (ρ) for ordinal outcomes; Mann–Whitney *U*: Cohen’s *d* with mean ± SD age when the variant was present vs. absent; Kruskal–Wallis *H*: ε^2^ for nominal outcomes. BH and BY, Benjamini–Hochberg and Benjamini–Yekutieli FDR-adjusted *p* values. FDR, false discovery rate; IFAC, International Frontal Sinus Anatomy Classification; L, left; PA, posteroanterior; R, right; SD, standard deviation.

**Table A4:** Sex-related associations: all 30 tests (*n* = 88).

Outcome	Test	Effect Size (95% CI)	*p*	BH	BY
R temporal pneumatisation	Mann–Whitney *U*	*r*_rb_ = 0.13 (M vs. F)	0.273	0.533	1.000
L temporal pneumatisation	Mann–Whitney *U*	*r*_rb_ = −0.06 (M vs. F)	0.617	0.888	1.000
R frontal sinus size	Mann–Whitney *U*	*r*_rb_ = 0.17 (M vs. F)	0.132	0.398	1.000
L frontal sinus size	Mann–Whitney *U*	*r*_rb_ = 0.26 (M vs. F)	0.017	0.165	0.660
R IFAC agger nasi	Fisher exact	OR = 0.88 (0.33–2.41); M 66.7% vs. F 69.4%	0.821	0.923	1.000
R IFAC supra-agger	Fisher exact	OR = 1.32 (0.38–4.54); M 20.5% vs. F 16.3%	0.782	0.923	1.000
R IFAC supra-bulla	Fisher exact	OR = 0.89 (0.34–2.30); M 35.9% vs. F 38.8%	0.827	0.923	1.000
R IFAC supraorbital ethmoidal	Fisher exact	OR = 3.51 (1.20–11.06); M 41.0% vs. F 16.3%	0.015	0.165	0.660
L IFAC agger nasi	Fisher exact	OR = 0.65 (0.23–1.77); M 64.1% vs. F 73.5%	0.363	0.640	1.000
L IFAC supra-agger	Fisher exact	OR = 0.59 (0.12–2.43); M 10.3% vs. F 16.3%	0.537	0.848	1.000
L IFAC supra-agger frontal	Fisher exact	OR = 1.94 (0.21–24.42); M 7.7% vs. F 4.1%	0.652	0.888	1.000
L IFAC supra-bulla	Fisher exact	OR = 1.12 (0.44–2.86); M 43.6% vs. F 40.8%	0.830	0.923	1.000
L IFAC supra-bulla frontal	Fisher exact	OR = 0.83 (0.07–7.65); M 5.1% vs. F 6.1%	1.000	1.000	1.000
L IFAC supraorbital ethmoidal	Fisher exact	OR = 2.35 (0.89–6.40); M 46.2% vs. F 26.5%	0.073	0.398	1.000
Frontal septal cell	Fisher exact	OR = 3.69 (1.21–12.31); M 38.5% vs. F 14.3%	0.013	0.165	0.660
Ethmoid septal spur	Fisher exact	OR = 2.24 (0.87–5.95); M 66.7% vs. F 46.9%	0.085	0.398	1.000
Ethmoid pneumatised middle turbinate	Fisher exact	OR = 0.61 (0.24–1.57); M 51.3% vs. F 63.3%	0.284	0.533	1.000
Ethmoid paradoxical middle turbinate	Fisher exact	OR = 0.33 (0.05–1.41); M 7.7% vs. F 20.4%	0.133	0.398	1.000
Ethmoid pneumatised superior turbinate	Fisher exact	OR = 2.00 (0.64–6.52); M 28.2% vs. F 16.3%	0.201	0.465	1.000
Ethmoid pneumatised uncinate process	Fisher exact	OR = 2.76 (0.54–18.25); M 15.4% vs. F 6.1%	0.177	0.442	1.000
Ethmoid suprabullar cell	Fisher exact	OR = 1.14 (0.45–2.90); M 46.2% vs. F 42.9%	0.830	0.923	1.000
Ethmoid agger nasi cell	Fisher exact	OR = 1.12 (0.41–3.17); M 71.8% vs. F 69.4%	1.000	1.000	1.000
Ethmoid Onodi cell	Fisher exact	OR = 0.38 (0.14–1.00); M 28.2% vs. F 51.0%	0.049	0.366	1.000
Ethmoid Haller cell	Fisher exact	OR = 0.55 (0.19–1.49); M 25.6% vs. F 38.8%	0.255	0.533	1.000
Ethmoid pneumatised crista galli	Fisher exact	OR = 1.94 (0.21–24.42); M 7.7% vs. F 4.1%	0.652	0.888	1.000
Ethmoid supraorbital ethmoid cell	Fisher exact	OR = 2.13 (0.82–5.66); M 48.7% vs. F 30.6%	0.123	0.398	1.000
Ethmoid lamina papyracea dehiscence	Fisher exact	OR = 0.94 (0.13–5.94); M 7.7% vs. F 8.2%	1.000	1.000	1.000
R sphenoid lateral type	Fisher–Freeman–Halton	*V* = 0.27	0.154	0.419	1.000
L sphenoid lateral type	Fisher–Freeman–Halton	*V* = 0.28	0.125	0.398	1.000
Sphenoid posteroanterior type	Fisher–Freeman–Halton	*V* = 0.20	0.498	0.830	1.000

Mann–Whitney *U*: rank-biserial correlation (*r*_rb_; positive values indicate larger values in men); Fisher exact: conditional maximum-likelihood OR with exact 95% CI (men vs. women) and prevalence in men (M) and women (F); Fisher–Freeman–Halton: Monte Carlo *p* value (20,000 replicates) with Cramér’s *V*. BH and BY, Benjamini–Hochberg and Benjamini–Yekutieli FDR-adjusted *p* values. CI, confidence interval; FDR, false discovery rate; IFAC, International Frontal Sinus Anatomy Classification; L, left; OR, odds ratio; PA, posteroanterior; R, right.

**Table A5:** Squared correlation ratios (η^2^) between the variables and the first three dimensions of the multiple correspondence analysis.

Variable	Dimension 1	Dimension 2	Dimension 3
R temporal pneumatisation	0.127	0.414	0.307
L temporal pneumatisation	0.119	0.487	0.256
R frontal sinus size	0.151	0.210	0.140
L frontal sinus size	0.052	0.198	0.091
R IFAC agger nasi	0.402	0.037	0.034
L IFAC agger nasi	0.296	0.098	0.053
R IFAC supra-bulla	0.601	0.053	0.007
L IFAC supra-bulla	0.561	0.082	0.006
R IFAC supraorbital ethmoidal	0.071	0.265	0.222
L IFAC supraorbital ethmoidal	0.030	0.157	0.373
Pneumatised middle turbinate	0.080	0.045	0.223
Onodi cell	0.023	0.003	0.017

η^2^, squared correlation ratio between each variable and each dimension. IFAC, International Frontal Sinus Anatomy Classification; L, left; MCA, multiple correspondence analysis; R, right.
